# Supplementary material for: A new hERG allosteric modulator rescues genetic and drug‐induced long‐QT syndrome phenotypes in cardiomyocytes from isogenic pairs of patient induced pluripotent stem cells
Source: EMBO Mol Med. 2016 Jul 28;8(9):1065–81. doi: 10.15252/emmm.201606260 (PMC5009811; doi:10.15252/emmm.201606260)
Supplement: Supplementary file 1 — Appendix [file EMMM-8-1065-s001.pdf]

Appendix Figures

A new hERG allosteric modulator rescues genetic and drug-induced Long-QT Syndrome phenotypes in cardiomyocytes from isogenic pairs of patient induced pluripotent stem cells

Luca Sala, Zhiyi Yu, Dorien Ward-van Oostwaard, Jacobus P.D. van Veldhoven, Alessandra Moretti, Karl-Ludwig Laugwitz, Christine L. Mummery, Adriaan P. IJzerman, Milena Bellin*

Table of Contents:

Appendix Figure S1
Appendix Figure S2
Appendix Figure S3
Appendix Figure S4
Appendix Table S1
Appendix Table S2

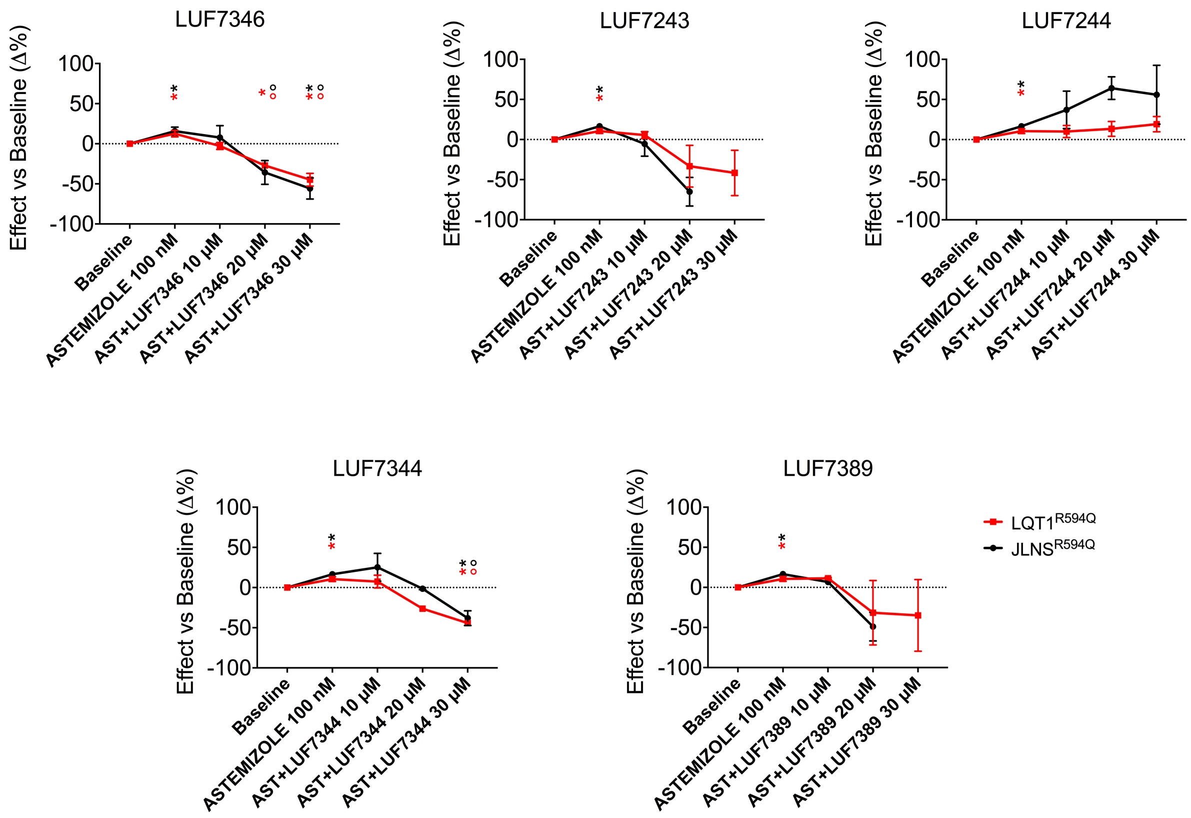

Appendix Figure S1: Effect of allosteric modulators on QT interval measured in hiPSC-CMs.

Pilot experiments on MEA displaying the effect of different LUF compounds in drug-induced LQTS. * = $p < 0.05$ vs respective baseline. ° = $p < 0.05$ vs AST. The colour of the symbols indicates the relative statistical significance. N: 3-10.

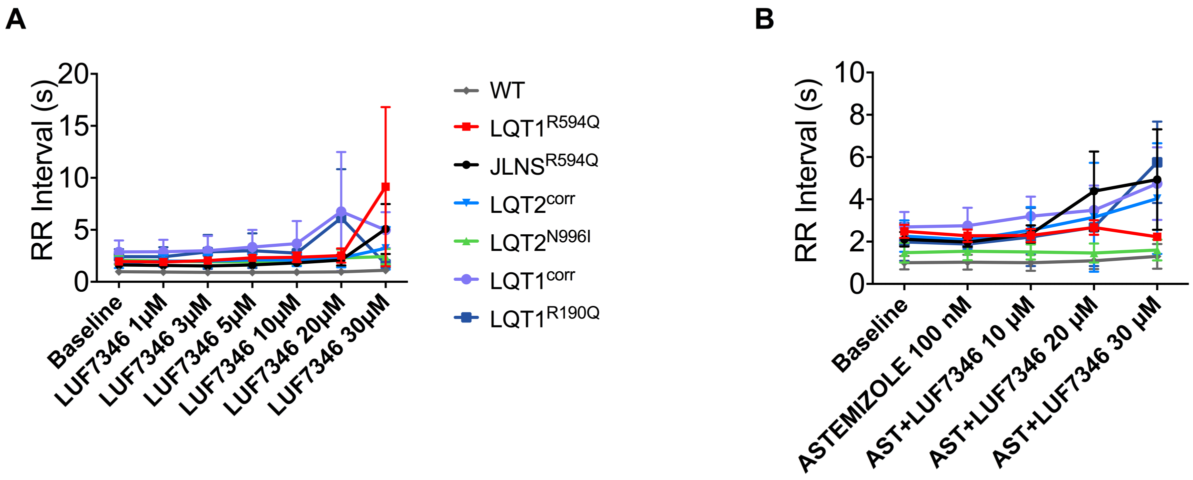

Appendix Figure S2: LUF7346 does not alter beating frequency on spontaneously beating hiPSC-CMs monolayers. A) Average effects of increasing concentrations of LUF7346 on the RR interval measured with MEA. N: 14-37. B) Average effects of AST and increasing concentrations of LUF7346 in the presence of AST, on the RR interval measured with MEA. N: 7-14.

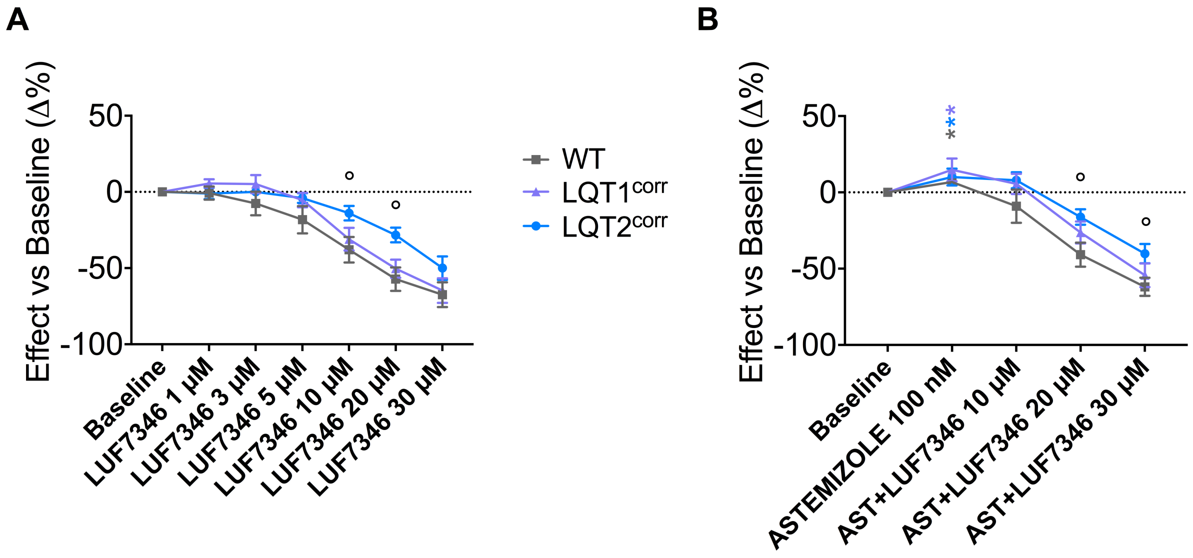

Appendix Figure S3: Comparison of LUF7346 effect on QT interval of control hiPSC-CMs. A) Average effects of increasing concentrations of LUF7346 on the QT interval measured with MEA. N: 8-17. ° = $p < 0.05$ vs WT and LQT1^{corr}. B) Average effects of AST and increasing concentrations of LUF7346 in the presence of AST on the QT interval measured with MEA. * = $p < 0.05$ vs respective baseline. The colour of the asterisks indicates comparisons and relative statistical significance. ° = $p < 0.05$ vs WT and LQT1^{corr}. N: 6-9.

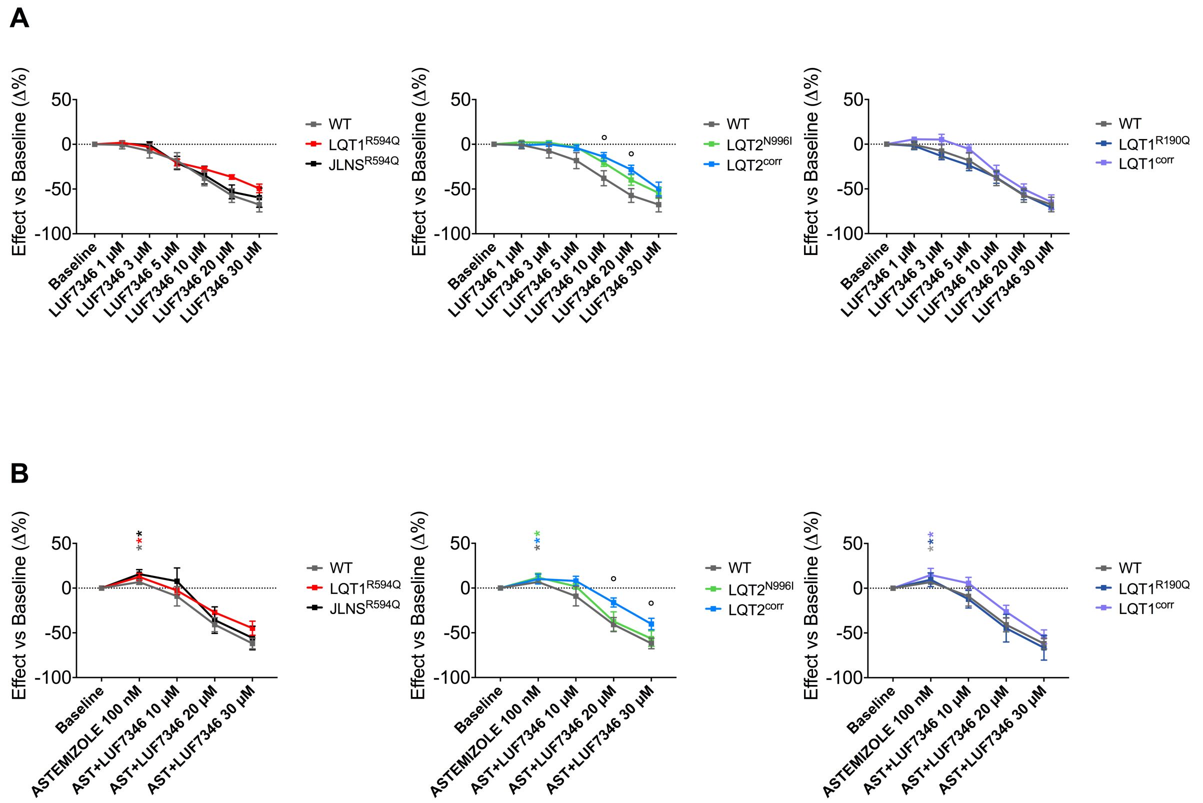

Appendix Figure S4: Comparison of LUF7346 effect among isogenic hiPSC lines.

A) Average of the effect of increasing concentrations of LUF7346 on QT interval duration compared to baseline. ° = $p < 0.05$ vs WT. The colour of the symbols indicates comparisons and relative statistical significance. N: 7-14. B) Average data of the effect of AST and increasing concentrations of LUF7346 in the presence of AST on QT interval durations, compared to baseline. * = $p < 0.05$. The colour of the symbols indicates the relative statistical significance. N: 7-12.

Appendix Table S1 - The percentage specific binding of [^{3}H]dofetilide to the hERG channel after 6 min of dissociation in the absence ($B_{control}$) or presence of 10 or 50 µM of the indicated compounds (B). Values are means (±SEM) of at least three independent experiments performed in duplicate.

**Compounds**	**%B/$B_{control}$**	
	**10 µM**	**50 µM**
**Control**	100	100
**+LUF7243**	71±2	23±3
**+LUF7244**	43±2	38±5
**+LUF7344**	47±2	28±1
**+LUF7346**	44±1	16±0.01
**+LUF7389**	38±3	26±3
**n**	>3	>3

Appendix Table S2- Parameters from Major-axis regression analysis in all hiPSC-CMs analysed.

WT			
	**Uncorrected**	**Bazett's formula**	**Fridericia's formula**
**R^2**	0.2679	0.5189	0.4508
**1/slope**	-0.01197	-0.006264	-0.007484
**Deviation from 0**	NS	*	*
**p-value**	0.1535	0.0284	0.0477
**$LQT1^{R594Q}$**			
	**Uncorrected**	**Bazett's formula**	**Fridericia's formula**
**R^2**	0.05125	0.279	0.0939
**1/slope**	0.07485	-0.04008	-0.06946
**Deviation from 0**	NS	*	*
**p-value**	0.0904	<0.0001	0.0204
**$JLNS^{R594Q}$**			
	**Uncorrected**	**Bazett's formula**	**Fridericia's formula**
**R^2**	0.3157	0.2306	0.1037
**1/slope**	0.107	-0.0261	-0.03653
**Deviation from 0**	NS	*	NS
**p-value**	0.578	0.0035	0.0592
**$LQT2^{corr}$**			
	**Uncorrected**	**Bazett's formula**	**Fridericia's formula**
**R^2**	0.1035	0.3587	0.2824
**1/slope**	-0.0265	-0.01516	-0.01685
**Deviation from 0**	NS	*	*
**p-value**	0.2422	0.0183	0.0415
**$LQT2^{N996I}$**			
	**Uncorrected**	**Bazett's formula**	**Fridericia's formula**
**R^2**	0.001487	0.1312	0.06852
**1/slope**	-0.1356	-0.01798	-0.02317
**Deviation from 0**	NS	NS	NS
**p-value**	0.8613	0.082	0.2166
**$LQT1^{corr}$**			

	Uncorrected	Bazett's formula	Fridericia's formula
**R^2**	0.04683	0.004937	0.01185
**1/slope**	-0.01909	-0.102	0.1919
**Deviation from 0**	NS	NS	NS
**p-value**	0.4574	0.8113	0.9151
**LQT1^{R190Q}**			
	**Uncorrected**	**Bazett's formula**	**Fridericia's formula**
**R^2**	0.2733	0.01581	0.1194
**1/slope**	0.004213	0.05819	0.009301
**Deviation from 0**	NS	NS	NS
**p-value**	0.099	0.4749	0.2979
