# Supplementary material for: A new hERG allosteric modulator rescues genetic and drug‐induced long‐QT syndrome phenotypes in cardiomyocytes from isogenic pairs of patient induced pluripotent stem cells
Source: EMBO Mol Med. 2016 Jul 28;8(9):1065–81. doi: 10.15252/emmm.201606260 (PMC5009811; doi:10.15252/emmm.201606260)
Supplement: Supplementary file 2 — Expanded View Figures PDF [file EMMM-8-1065-s002.pdf]

Expanded View Figures

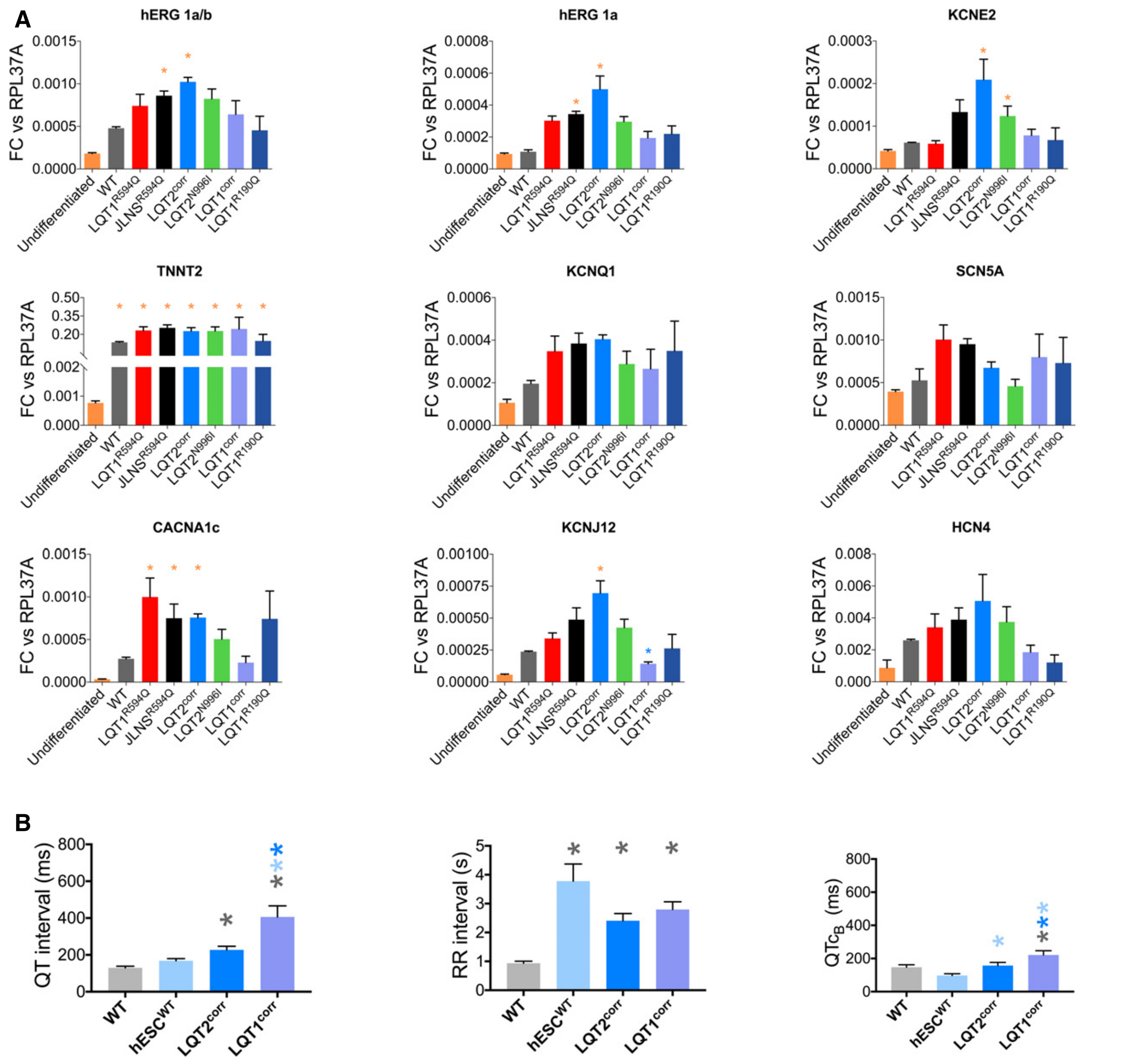

Figure EV1. Gene expression analysis and QT interval under baseline conditions.

A Expression analysis as measured by RT–qPCR of cardiac ion channel genes and sarcomeric protein *TNNT2* gene in undifferentiated hiPSCs and hiPSC-CMs. Data are expressed as fold change versus. *RPL37A*. $N = 3$. $*P < 0.05$. The colour of the asterisks indicates comparisons and relative statistical significance.

B QT intervals (left), RR intervals (right) and QTcB intervals measured with MEA in CMs derived from all the control hPSC lines under baseline conditions. $N = 17$. $*P < 0.05$. The colour of the asterisks indicates comparisons and relative statistical significance.

Data Information: (A) Kruskal–Wallis test with Dunn's multiple comparisons test: hERG 1a/b: JLNS^{R594Q} versus Undiff.: 0.0296; LQT2^{corr} versus Undiff.: 0.0011. hERG 1a: JLNS^{R594Q} versus Undiff.: 0.0088; LQT2^{corr} versus Undiff.: 0.0012. KCNE2: LQT2^{corr} versus Undiff.: 0.0012; LQT2^{N996I} versus Undiff.: 0.0468. TNNT2: WT versus Undiff.: 0.0458; LQT1^{R594Q} versus Undiff.: 0.0320; JLNS^{R594Q} versus Undiff.: 0.0052; LQT2^{corr} versus Undiff.: 0.0444. LQT2^{N996I} versus Undiff.: 0.0468. LQT1^{corr} versus Undiff.: 0.0444. LQT1^{R190Q} versus Undiff.: 0.0493. CACNA1c: LQT1^{R594Q} versus Undiff.: 0.0076; JLNS^{R594Q} versus Undiff.: 0.0429; LQT2^{corr} versus Undiff.: 0.0164. KCNJ12: LQT2^{corr} versus Undiff.: 0.0024; LQT2^{corr} versus LQT1^{corr}: 0.0060. (B) One-way ANOVA with Holm–Sidak's multiple comparison test: QT intervals: WT versus LQT1^{corr}: < 0.0001; LQT2^{corr} versus LQT1^{corr}: 0.0006; LQT1^{corr} versus hESC^{WT}: < 0.0001. RR intervals: WT versus LQT1^{corr}: < 0.0001; WT versus LQT2^{corr}: 0.0009; WT versus hESC^{WT}: < 0.0001. QTc$_B$: WT versus LQT1^{corr}: 0.0475; LQT1^{corr} versus LQT2^{corr}: 0.0432; LQT1^{corr} versus hESC^{WT}: < 0.0001. LQT2^{corr} versus hESC^{WT}: 0.0432. (A, B) Data are expressed and plotted as the mean ± SEM.

Source data are available online for this figure.

 © 2016 The Authors

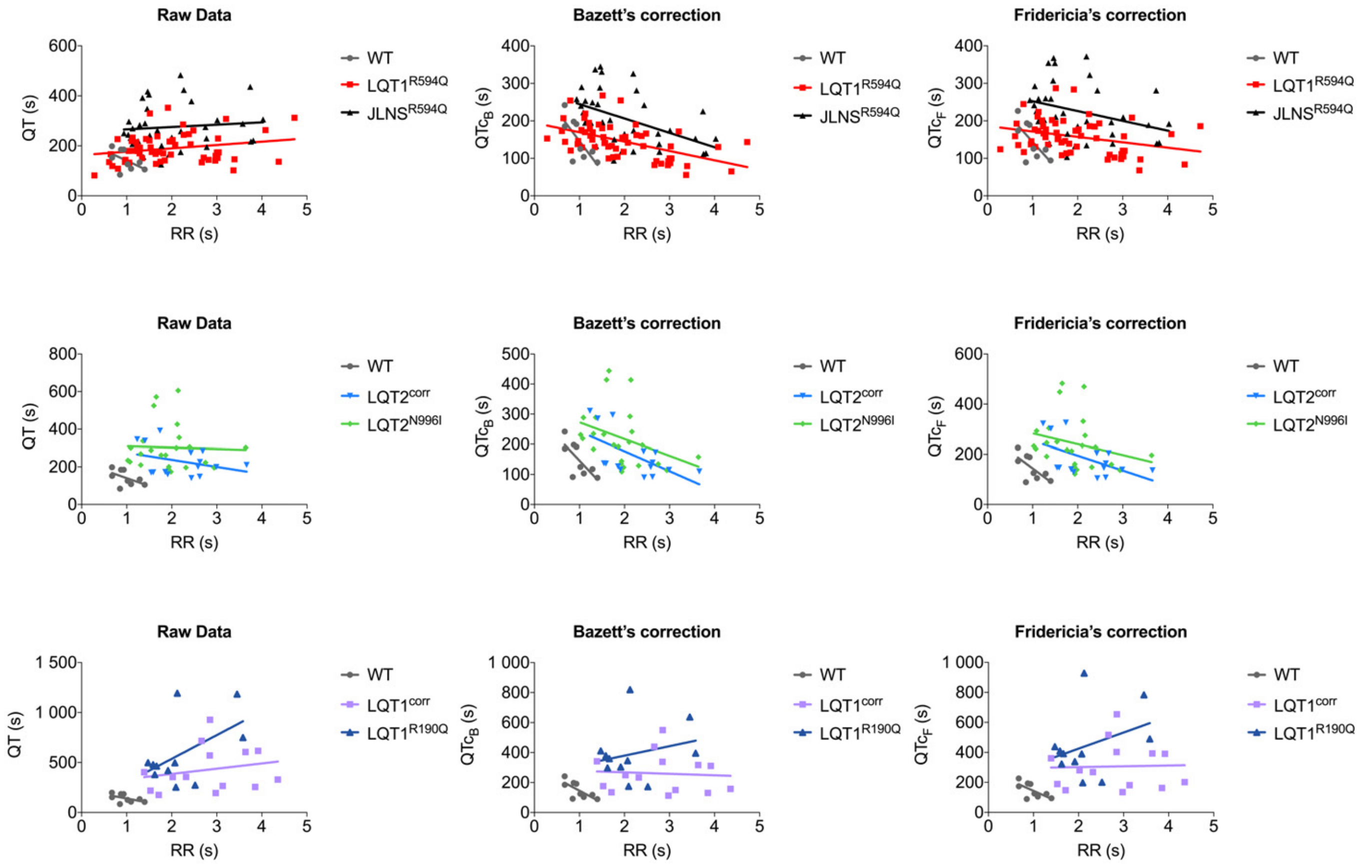

Figure EV2. Major-axis regression analysis on the relationship between QT and RR intervals.

Correlation between QT interval (left), QT interval corrected with Bazett's formula (QTc_B, middle), QT corrected with Fridericia's formula (QTc_F, right) and RR intervals. Bar graphs are divided by isogenic pairs (LQT1^{R594Q} and JLNS^{R594Q}, top; LQT2^{corr} and LQT2^{N996I}, centre; LQT1^{corr} and LQT1^{R190Q}, bottom), and in each graph, the unrelated WT is shown as a comparison.

© 2016 The Authors

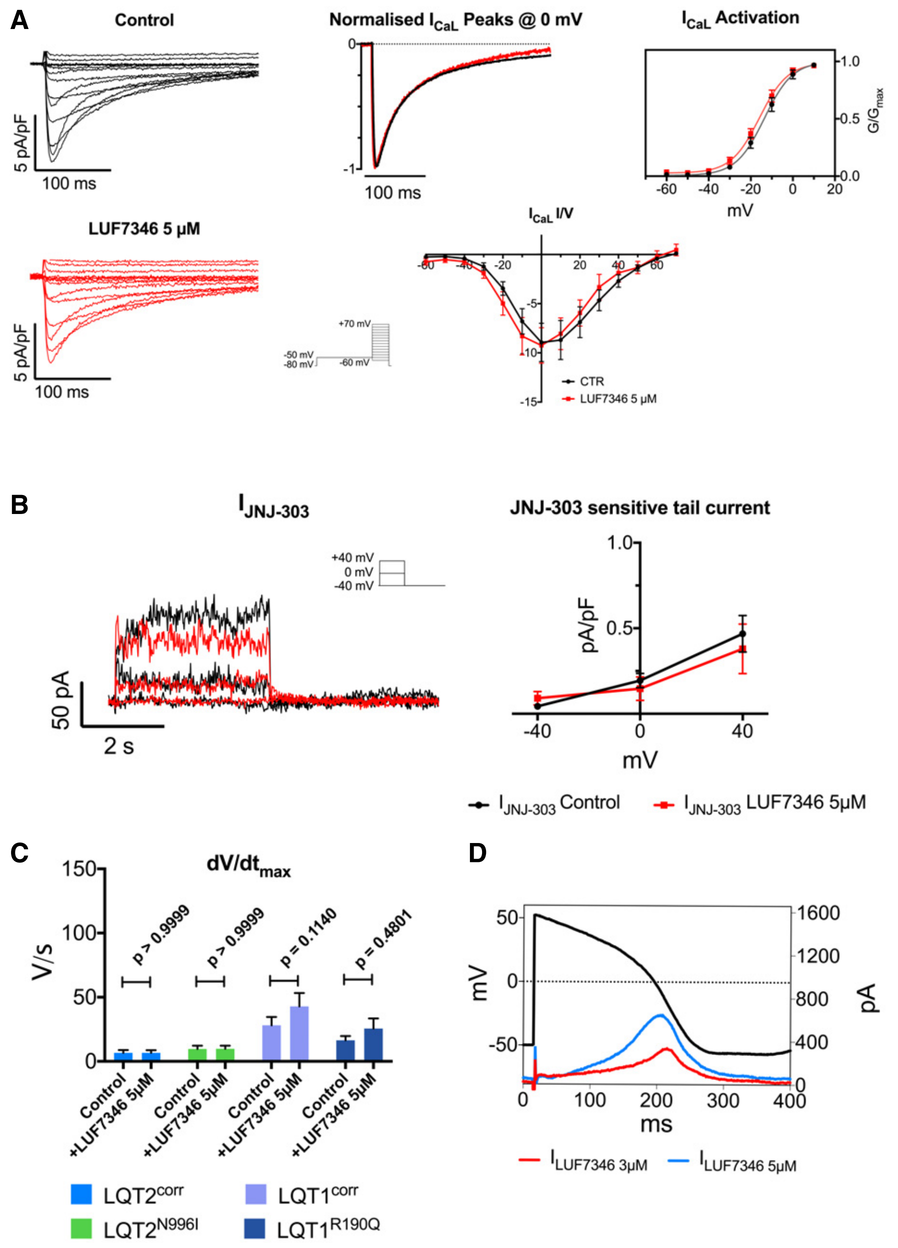

Figure EV3. Specificity of LUF7346.

A Representative I_{CaL} traces in control (black) and after the addition of 5 µM LUF7346 (red), normalised I_{CaL} peaks at 0 mV, steady-state I_{CaL} activation and current/voltage relationship (I/V) demonstrate that 5 µM LUF7346 has no effect on I_{CaL} in WT hiPSC-CMs. Inset: voltage-clamp protocol. *N* = 8, 6.

B Representative I_{Ks} traces and JNJ-303-sensitive tail currents at -40 mV in control (black) and in presence of 5 µM LUF7346 (red) demonstrate that 5 µM LUF7346 has no effect on I_{Ks} in WT hiPSC-CMs. Inset: voltage-clamp protocol. *N* = 9, 7.

C LUF7346 5 µM has no effect on upstroke velocity, as measured in LQT2^{corr}-, LQT2^{N996I}-, LQT1^{corr}- and LQT1^{R190Q}-CMs; *N* = 12, 11, 13, 16, respectively. A repeated-measures two-way ANOVA with Sidak's multiple comparisons tests was performed to assess the statistical significance.

D AP Clamp measurements showing the current induced by LUF7346 at 3 µM ($I_{LUF7346}$ 3 µM, red) and 5 µM ($I_{LUF7346}$ 5 µM, blue).

Data information: (A–C) Data are expressed and plotted as the mean ± SEM.

 © 2016 The Authors

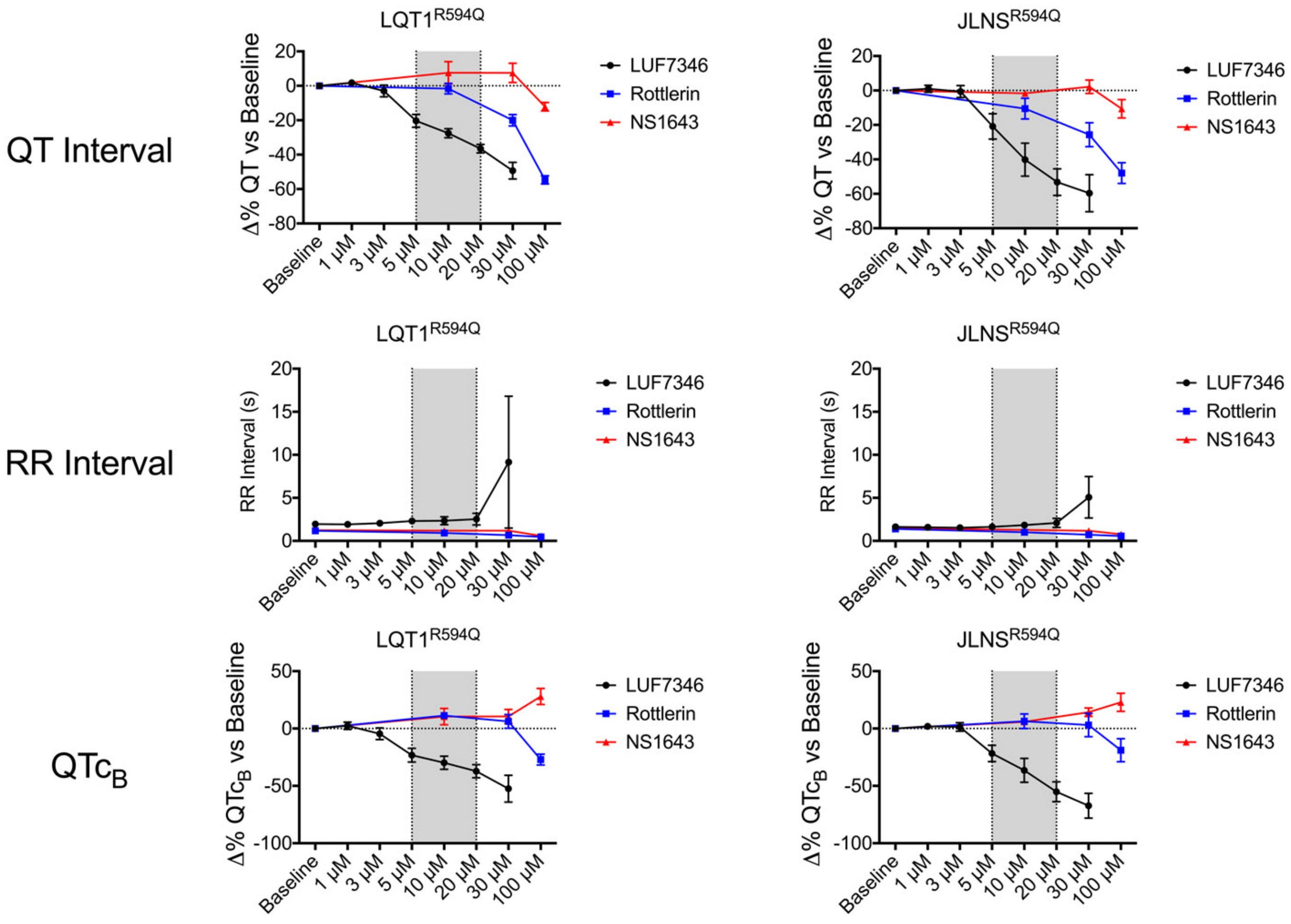

Figure EV4. Comparison of LUF7346 with known hERG modulators.

QT interval (top), RR interval (centre) and QTc_B interval (bottom) were measured at increasing concentrations of LUF7346, Rottlerin, and NS1643 on MEA. $LQT1^{R594Q}$- (left) and $JLNS^{R594Q}$-CMs (right) were used. Data are expressed as delta percentage compared to the baseline. The grey window highlights the most biologically relevant concentration in which LUF7346 has stronger effect than the other two tested compounds. $N = 5–8$. Data are expressed and plotted as the mean ± SEM.

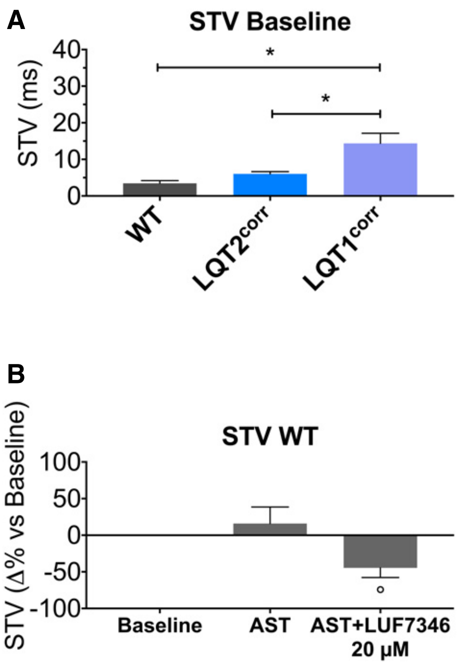

Figure EV5. Chronic effect of LUF7346 on hESC-CMs.

A STV values calculated on spontaneously beating hiPSC-CM clusters over 30 consecutive beats as measured with MEA. N = 5–19. *P < 0.05.

B STV changes calculated over 30 consecutive beats in the presence of AST and AST+LUF7346 20 μM in WT-CMs. °P < 0.05 versus AST. N = 5.

Data information: (A, B) Unpaired one-way ANOVA with Holm–Sidak's multiple comparisons test. Data are expressed and plotted as the mean ± SEM.

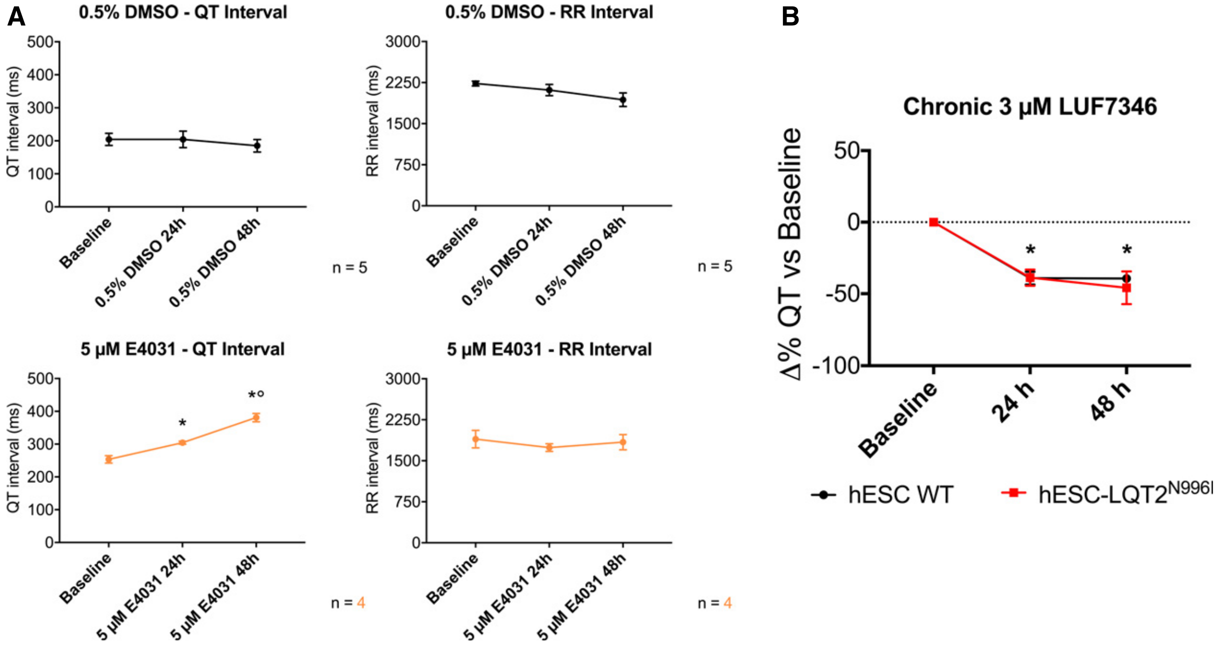

Figure EV6. Chronic effect of LUF7346 on hESC-CMs.

A Effect of positive (5 μM E4031, bottom) and negative (0.5% DMSO (top) controls on QT (left) and RR (right) intervals in hESC-LQT2^{N996I}-CMs, as measured on multi-well MEAs. *P < 0.05 versus baseline. °P < 0.05 versus 24 h. N = 5 and 4 for positive and negative controls, respectively.

B Chronic effect of 3 μM LUF7346 on WT- and hESC-LQT2^{N996I}-CMs, as measured on multi-well MEAs. *P < 0.05 versus baseline. N = 10 and 8 for WT-hESC and LQT2^{N996I}-hESC-CMs, respectively.

Data Information: (A) Paired one-way ANOVA with Holm–Sidak's multiple comparisons test. 24 h versus baseline = 0.0276; 48 h versus baseline 0.0003 versus baseline. 48 h versus 24 h: P = 0.0042. (B) Two-ways ANOVA with Holm–Sidak's multiple comparisons test. P < 0.0001 versus the respective baseline. (A, B) Data are expressed and plotted as the mean ± SEM.

 © 2016 The Authors
