# Supplementary material for: A new hERG allosteric modulator rescues genetic and drug‐induced long‐QT syndrome phenotypes in cardiomyocytes from isogenic pairs of patient induced pluripotent stem cells
Source: EMBO Mol Med. 2016 Jul 28;8(9):1065–81. doi: 10.15252/emmm.201606260 (PMC5009811; doi:10.15252/emmm.201606260)
Supplement: Supplementary file 3 — Source Data for Expanded View [file EMMM-8-1065-s003.zip › EMM-2016-06260_SourceData_EV1A/Readme.rtf]

Representative Movies recorded from beating monolayers at day 14 of differentiation in the following lines:1 - WT.mp4: Referring to WT line in Figure 2A and Figure EV1A2 - LQT1^R594Q.mp4: Referring to LQT1R594Q line in Figure 2A and Figure EV1A3 - JLNS^R594Q.mp4: Referring to JLNSR594Q line in Figure 2A and Figure EV1A4 - LQT2^corr.mp4: Referring to LQT2corr line in Figure 2A and Figure EV1A5 - LQT2^N996I.mp4: Referring to LQT2N996I line in Figure 2A and Figure EV1A6 - LQT1^corr.mp4: Referring to LQT1corr line in Figure 2A and Figure EV1A7 - LQT1^R190Q.mp4: Referring to LQT1R190Q line in Figure 2A and Figure EV1A
